# Performance in myoelectric pattern recognition improves with transcranial direct current stimulation

**DOI:** 10.1038/s41598-024-62185-x

**Published:** 2024-05-23

**Authors:** Shahrzad Damercheli, Kelly Morrenhof, Kirstin Ahmed, Max Ortiz-Catalan

**Affiliations:** 1 Center for Bionics and Pain Research, Mölndal, Sweden; 2 Department of Electrical Engineering, Chalmers University of Technology, Gothenburg, Sweden; 3 Bionics Institute, Melbourne, Australia; 4 Medical Bionics Department, University of Melbourne, Melbourne, Australia; 5 NeuroBioniX, Melbourne, Australia; 6 Prometei Pain Rehabilitation Center, Vinnytsia, Ukraine

**Keywords:** Biomedical engineering, Electrical and electronic engineering, Translational research

## Abstract

Sensorimotor impairments, resulting from conditions like stroke and amputations, can profoundly impact an individual’s functional abilities and overall quality of life. Assistive and rehabilitation devices such as prostheses, exo-skeletons, and serious gaming in virtual environments can help to restore some degree of function and alleviate pain after sensorimotor impairments. Myoelectric pattern recognition (MPR) has gained popularity in the past decades as it provides superior control over said devices, and therefore efforts to facilitate and improve performance in MPR can result in better rehabilitation outcomes. One possibility to enhance MPR is to employ transcranial direct current stimulation (tDCS) to facilitate motor learning. Twelve healthy able-bodied individuals participated in this crossover study to determine the effect of tDCS on MPR performance. Baseline training was followed by two sessions of either sham or anodal tDCS using the dominant and non-dominant arms. Assignments were randomized, and the MPR task consisted of 11 different hand/wrist movements, including rest or no movement. Surface electrodes were used to record EMG and the MPR open-source platform, BioPatRec, was used for decoding motor volition in real-time. The motion test was used to evaluate performance. We hypothesized that using anodal tDCS to increase the excitability of the primary motor cortex associated with non-dominant side in able-bodied individuals, will improve motor learning and thus MPR performance. Overall, we found that tDCS enhanced MPR performance, particularly in the non-dominant side. We were able to reject the null hypothesis and improvements in the motion test’s completion rate during tDCS (28% change, p-value: 0.023) indicate its potential as an adjunctive tool to enhance MPR and motor learning. tDCS appears promising as a tool to enhance the learning phase of using assistive devices using MPR, such as myoelectric prostheses.

## Introduction

Sensorimotor impairments, such as those arising from stroke and amputations, have significant effects on functional capabilities and quality of life. In one study (n = 94,905), 21% of stroke patients experienced motor impairment alone and in total, 82% experienced motor impairment in addition to sensory, cognitive, or sensory and cognitive impairment^1^. The most prevalent impairment following stroke is a contralateral upper limb hemiparesis affecting > 80% of patients during the acute phase and > 40% in the chronic phase^2^.

Amputations are one of the most extreme cases of sensorimotor impairments, resulting in reduced quality of life, and often unpleasant sensations and pain in the phantom limb^3,4^. Although there is no perfect solution for these impairments, advanced prosthetic systems can restore a certain degree of function after an amputation^5–7^, and phantom limb pain (PLP) can be alleviated by guided plasticity approaches such mirror therapy^8–11^, graded motor imagery^12,13^, and phantom motor execution^14,15^. Similar approaches have also been used for the rehabilitation of stroke patients^2^.

One method to increase prosthetic limb control is myoelectric pattern recognition (MPR) which decodes patterns of muscle activity in the residual muscles of amputated limbs^16,17^. Embedded systems running MPR are able to infer movement intention using machine learning algorithms to control prosthetic devices^18^. This provides a more intuitive control of the prosthetic limb compared to simpler one-to-one electromyography (EMG) strategies (one EMG signal to drive one prosthetic function). In addition, MPR has been employed for treatment of PLP in individuals with amputation^14,19^, and functional restoration in patients after stroke. One way to increase prosthetic control performance using MPR is using targeted rehabilitation that includes repetitive exercise and prolonged training. Research indicates that this also increases prosthetic use^20,21^, however, these methods are often time-consuming and can lead to user frustration and possibly abandonment of the prosthetic device.

Treatments that are used in stroke recovery include non-invasive brain stimulation techniques which modulate brain activity by introducing external stimuli^22^. Examples include transcranial electrical stimulation (tES), such as transcranial direct current stimulation (tDCS), and repetitive transcranial magnetic stimulation (rTMS). These techniques enhance or inhibit neuronal activity^23^ and consequently, alter sensorimotor and cognitive functions^24,25^. tES changes the cell membrane potential and thus modulate the spontaneous firing rate^22^, which has been shown to increase motor learning^26^ and the recovery of motor dysfunction^27^. A meta-analysis by Bai et al., concluded that tDCS is effective for stroke recovery of patients with motor dysfunction^27^. Furthermore tDCS has been used in the rehabilitation of several additional neurological disorders such as depression, anxiety, and schizophrenia^24,25,28^, as well as for the relief of PLP^29^.

In a small number of individuals with unilateral upper limb amputation, tDCS has been shown to improve the ability of the subjects to produce distinct EMG signals, which is useful for MPR applied to the control of prosthetic limbs^30^. Similarly, tDCS has shown promising results on hand performance in able-bodied individuals using the Jebsen Taylor hand function test (JTHFT). In one study, both the dominant and non-dominant primary motor cortex were targeted resulting in improved motor function in the non-dominant hand following modulation of the corresponding primary motor cortex^31^. The effect of tDCS seems to be observable in the affected or non-dominant side, however, the evidence is limited and replication by independent groups has not been conducted.

We undertake research in the field of prosthetic limbs^5,6^, rehabilitation of PLP^14,29^, and stroke^32^ which would all benefit from validating the efficacy of tDCS to improve MPR. Here, we evaluated the hypothesis that tDCS improves MPR in the non-dominant hand of able-bodied individuals in a cross-over, sham-controlled study. In addition, we also assess the effect of learning and the application of tDCS in the dominant arm. We used the completion rate of the motion test^33^ as a measure of the participants ability to accomplish a motor task using MPR. In addition, we also measured the time in which each task was completed (completion time), and the reliability of decoding (accuracy).

Twelve healthy able-bodied individuals conducted baseline training followed by two sessions of either sham or anodal tDCS on the primary motor cortex of the dominant and non-dominant arms, separately. Assignments were randomized, and the MPR task involved 11 different hand/wrist movements, including rest or no movement. Surface electrodes recorded EMG signals, and real-time motor volition decoding was performed using the open-source platform BioPatRec^17^. Performance evaluation was conducted using the motion test as implanted in BioPatRec.

## Result

### Participants

In total, 12 able-bodied individuals participated in this study, of which six were females and six were males ranging from 23–33 (27 ± 3.6) years old. Eleven participants were right-handed, one participant was left-handed. All study participants successfully completed all sessions, demonstrating good tolerance to the intervention. Upon initiation of anodal tDCS, all participants experienced a tingling sensation underneath the anode electrode of tDCS, attributed to the electrical current passing through the skin and underlying tissues^34^. One participant reported a side effect characterized by redness underneath the cathode electrode. This was a singular occurrence and did not necessitate termination of their participation in the study.

### Statistical analysis

**Figure 1:**
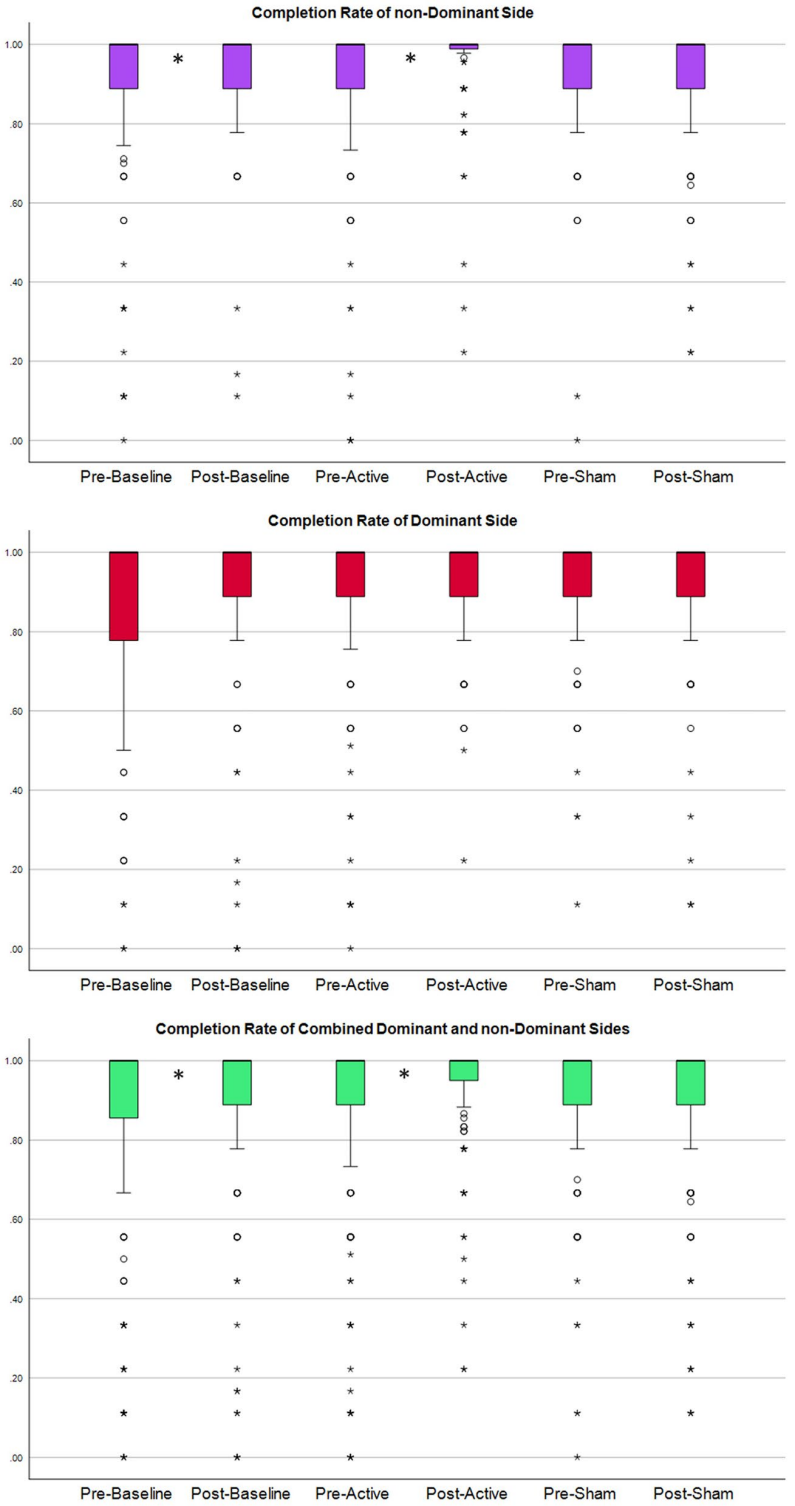
Displays the distribution of completion rate. Top: distribution of completion rate for combined movements of the ‘non-dominant side’. Middle and bottom plot: distributions of the completion rate for the ‘dominant sides’ and ‘combined dominant and non-dominant sides’, respectively. Columns 1 and 2 correspond to the baseline session without stimulation, before and after the break, respectively. Columns 3 and 4 represent the active tDCS session, column 3 depicting the completion rate before active stimulation and column 4 after. Columns 5 and 6 represent the sham tDCS session. An asterisk indicates a statistically significant difference between the two neighboring columns.

**Figure 2:**
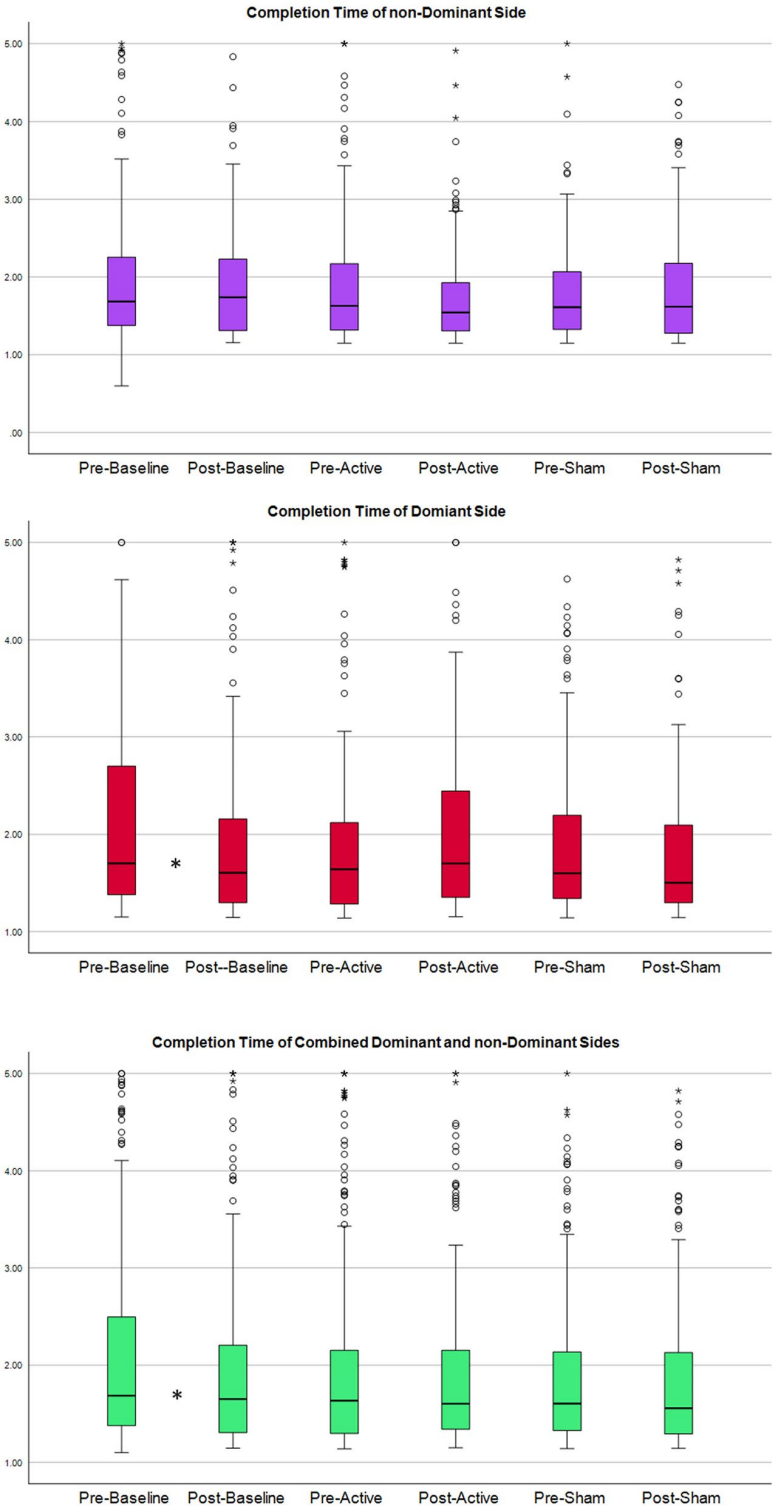
Displays the distribution of completion time. Top: distribution of completion time for combined movements of the ‘non-dominant side’. Middle and bottom plot: distributions of the completion time for the ‘dominant sides’ and ‘combined dominant and non-dominant sides’, respectively. Columns 1 and 2 correspond to the baseline session without stimulation, before and after the break, respectively. Columns 3 and 4 represent the active tDCS session, column 3 depicting the completion time before active stimulation and column 4 after. Columns 5 and 6 represent the sham tDCS session. An asterisk indicates a statistically significant difference between the two neighboring columns.

**Figure 3:**
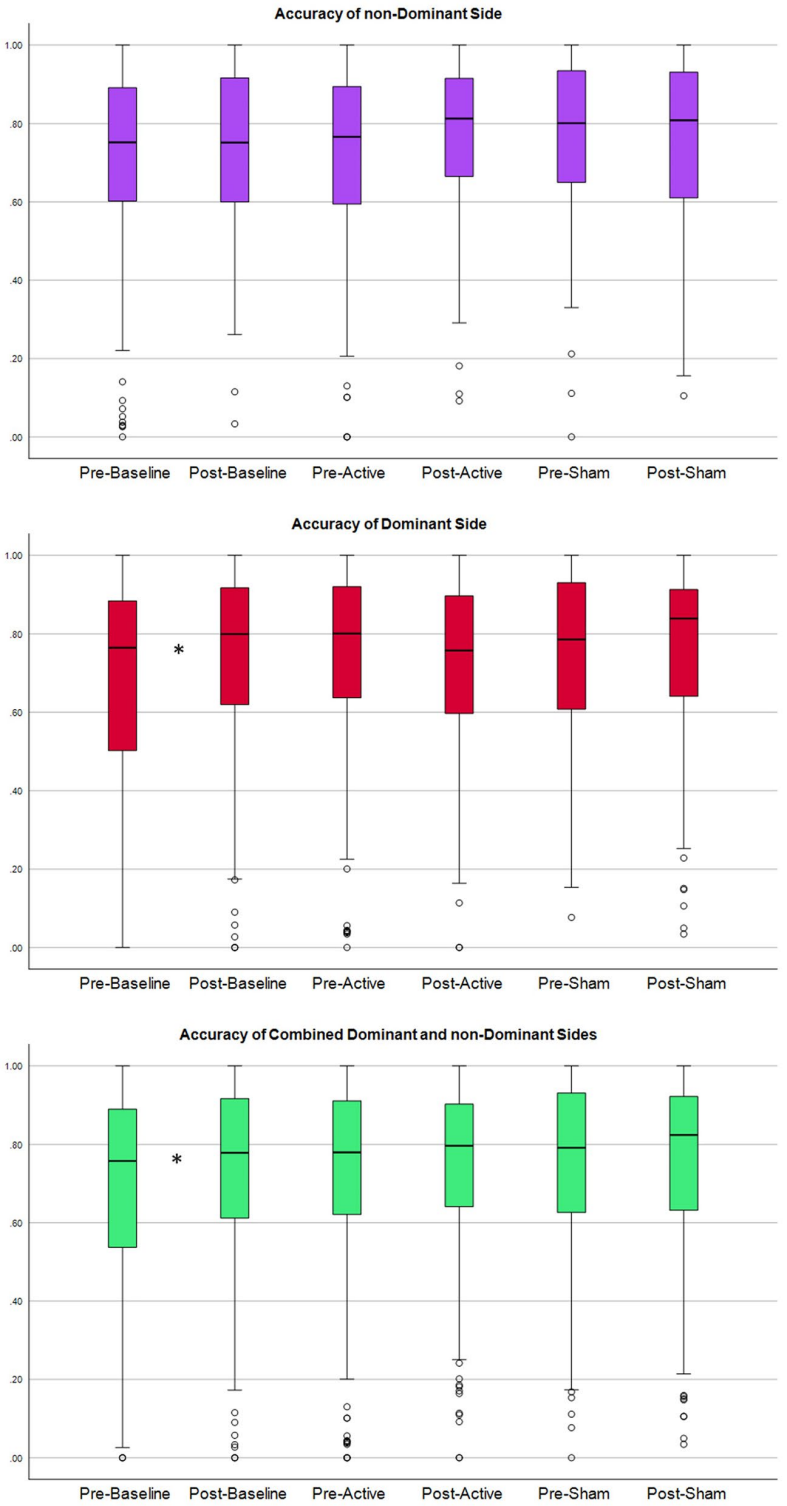
Displays the distribution of accuracy. Top: distribution of accuracy for combined movements of the ‘non-dominant side’. Middle and bottom plot: distributions of the accuracy for the ‘dominant sides’ and ‘combined dominant and non-dominant sides’, respectively. Columns 1 and 2 correspond to the baseline session without stimulation, before and after the break, respectively. Columns 3 and 4 represent the active tDCS session, column 3 depicting the accuracy before active stimulation and column 4 after. Columns 5 and 6 represent the sham tDCS session. An asterisk indicates a statistically significant difference between the two neighboring columns.

Due to the non-normal distribution of the data (tested by Shapiro–Wilkes and Kolmogorov) and the limited sample size, the non-parametric Wilcoxon Signed ranks test (WRST) (p-value < 0.05, two-tailed) was selected to investigate the significance of tDCS on completion rate, completion time, and accuracy (Table 1). The null hypothesis for this study was that there would be no significant changes in the completion rate before and after tDCS application. The analysis was conducted on data collected within each session, including baseline, sham stimulation, and active stimulation. Additionally, similar analyses were carried out for sub-groups based on dominant and non-dominant sides. Furthermore, we found no statistically significant differences between the participants performance after baseline and before active and sham sessions (Table 2). All participants completed three trials of the motion test, except for one participant who performed two trials during all visits and another participant who did only two trials during the baseline visit. Statistical results are presented in Table 1 and Table 2, and the distribution of the completion rate, completion time and accuracy in Figs. 1, 2, and 3 respectively.

**Table 1:** Results for MPR parameters, including completion rate, completion time, and accuracy.

Side	Pre vs post: within group	Completion rate			Completion time			Accuracy		
		p-values and percentage of change								
		Direction	Two-tailed	%	Direction	two-tailed	%	Direction	Two-tailed	%
Non-dominant side	Baseline session	Improvement	**0.0160**	**36.91**	Improvement	0.2304	12.36	Improvement	0.0730	96.83
	Active tDCS session	Improvement	**0.0236**	**27.34**	Improvement	0.1442	15.16	Improvement	0.1113	63.66
	Sham session	Deterioration	0.3307	4.47	Deterioration	0.2091	1.50	Deterioration	0.1384	9.30
Dominant side	Baseline session	Improvement	0.0708	19.85	Improvement	**0.0205**	**16.00**	Improvement	**0.0171**	**35.45**
	Active tDCS session	Improvement	0.1437	39.45	Deterioration	0.2323	1.94	Deterioration	0.4697	33.91
	Sham session	Improvement	0.9227	5.29	Improvement	0.2514	14.38	Improvement	0.3992	19.25
Combined dominant and non-dominant	Baseline session	Improvement	**0.0029**	**28.38**	Improvement	**0.0125**	**14.29**	Improvement	**0.0036**	**66.14**
	Active tDCS session	Improvement	**0.0126**	**33.40**	Improvement	0.8752	8.55	Improvement	0.5613	48.79
	Sham session	Deterioration	0.5503	4.88	Improvement	0.9698	7.94	Improvement	0.6856	14.27

Completion rate and accuracy improvements are defined as an increase in value. Completion time improvement is indicated by a decrease in the value.

Bold values indicates a statistically significant change.

**Table 2:** Two-tailed p-values from post-baseline to pre-active and pre-sham sessions.

Side	Post-baseline versus:	Completion rate	Completion time	Accuracy
		Two-tailed p-values		
Non-dominant side	Pre-active session	0.367	0.656	0.661
	Pre-sham session	0.455	0.164	0.057
Dominant side	Pre-active session	0.794	0.847	0.983
	Pre-sham session	0.156	0.684	0.495
Combined dominant and non-dominant	Pre-active session	0.702	0.631	0.710
	Pre-sham session	0.264	0.164	0.071

### Outcomes

In the baseline session, statistically significant improvements were observed in the completion rate (28% change, p-value: 0.0029), completion time (14% change, p-value: 0.0125), and accuracy (66% change, p-value: 0.0036). However, during the sham stimulation session, no significant change was detected in any of the MPR parameters. In contrast, the active stimulation session showed a significant improvement of 33% in the completion rate (p-value: 0.0126), but no significant change was detected in either completion time or accuracy.

### Subgroup Analysis

#### Non-dominant side

In the baseline session, a statistically significant improvement of 37% was observed in the completion rate (p-value: 0.0160). While, during the sham stimulation session, no statistically significant improvement was detected in any of the MPR parameters. In contrast, the active stimulation session showed a statistically significant improvement of 27% in the completion rate (p-value: 0.0236).

#### Dominant side

In the baseline session, statistically significant improvements were observed in the completion time (16% change, p-value: 0.0205), and accuracy (35% change, p-value: 0.0171), and substantial but not statistically significant improvement in completion rate (20% change, p-value: 0.0708). However, during the sham and active stimulation sessions, no statistically significant improvement was detected in any of the MPR parameters.

## Discussion

This study investigated the effect on the performance of MPR of a single session of anodal tDCS on the primary motor cortex. Testing on the non-dominant primary motor cortex assumes that disparities in the use of the dominant and non-dominant hands can imitate the differences observed between affected and intact hands in individuals with amputations, and the paretic and non-paretic hands in stroke patients. The relatively reduced dexterity of the non-dominant hand, as extensively demonstrated in the literature, stems from its asymmetric usage compared to the dominant hand^35^. Our findings showed a statistically significant improvement in the completion rate of the non-dominant side during the active tDCS day, but not during the sham tDCS day (27%, p = 0.02).

Our results demonstrated a statistically significant improvement in completion rate during the baseline training, but no further improvement during the sham session, which indicates that most learning could have taken place during the baseline training. Since there was no significant improvement in completion rate in the sham tDCS session, we can assume improvements during the active tDCS session are the result of motor excitability^26,36^ of the non-dominant motor cortex by anodal tDCS. Our findings are in line with previous work on MPR for prosthetics^30^ and motor performance in non-disable subjects^31^ and stroke patients^37^.

It is worth noting that the largest improvements were observed in the baseline session in which the participants were exposed to the MPR task for the first time (better performance in all metrics). This is not surprising and illustrates that the learning effect of practicing a task for the first time is higher than what is possible to achieve in a later single session of training with neuromodulation. Chronic neuromodulation will likely improve the MPR task further, and potentially show a change in all metrics as opposed to completion rate only. Completion rate relates to the ability of the participant to accomplish a task, whereas completion time and accuracy relate to how effectively it is achieved. Larger improvement in motor control will impact all three metrics, and whether this can be achieved faster with chronic neuromodulation has yet to be investigated.

On the dominant hand all three MPR metrics generated a non-negligible improvement during the baseline training, suggesting that skilled learning ceiling may have been achieved^26^. No further improvement was observed in either sham or active tDCS sessions. In a study by, Pan et al., decreased performance was observed in the unaffected arm in participants with unilateral amputation^30^.

Our findings support the previous literature and suggest that tDCS can enhance MPR performance, particularly in the non-dominant side. The improvements in the MPR we observed indicate the potential of tDCS as an adjunctive tool to enhance motor learning and performance in MPR. Our findings on able bodied participants are supported by the literature in other populations. For example Cho et al*.*, compared the effect of anodal tDCS over primary motor cortex combined with active or sham Mirror Therapy, and concluded that tDCS plus Mirror Therapy has a positive effect on the functional recovery of upper extremity in stroke patients^38^. Other works have compared the effects of active tDCS with sham tDCS on functional motor and somatosensory functions in acute stroke patients and demonstrated significant improvements using active tDCS (measured by Wolf motor function test and the Semmes Weinstein monofilament test)^39^.

The clinical significance of improving the learning phase in individuals using MPR controlled prosthetic limbs is faster rehabilitation time and potentially better functional outcomes with a reduced prosthetic abandonment rate. In addition, with respect to clinicians, the significance of using tDCS could be reduced consultation time. Moreover, the effectiveness and efficiency of MPR-based PLP treatments, such as Phantom motor execution, can be enhanced^29,40,41^.

We conducted our study on able-bodied participants, the next step is to translate this method to relevant populations such as stroke patients and individuals with limb loss, in prospective investigations with larger sample sizes and patient matched populations. This was an acute application of tDCS and the chronic effects should be further investigated.

## Conclusion

Restoration of function and rehabilitation of pain are of considerable importance after traumatic injuries and stroke. Decoding of motor volition via MPR is a promising tool that is now being used in different assistive and rehabilitation devices, and here we have provided further evidence that tDCS can facilitate MPR and thus potentially improve the clinical outcomes of patients using MPR. Further prospective investigations with larger sample sizes and matched patient populations (*e.g.,* limb loss or stroke) are necessary to produce higher quality evidence supporting this approach.

## Method

### Participants

The study was approved by the governing ethical committee in Sweden (approval number 2022-00883-02) and was performed in accordance with declaration of Helsinki and the relevant guidelines and regulations. The study was conducted using a double-blinded, randomized and sham-controlled design, wherein participants were unaware of the placebo control. Twelve healthy, able-bodied participants without prior tDCS experience were recruited in this study. When laterality was unclear, the Waterloo handedness questionnaire^42^ was used to assess the dominant side of the participant. Those with a history of neurological disorders or contradictions to tDCS were excluded from the study^34^. All participants received detailed information and provided written informed consent, including the consent to publish Fig. 6.

### Study design

**Figure 4:**
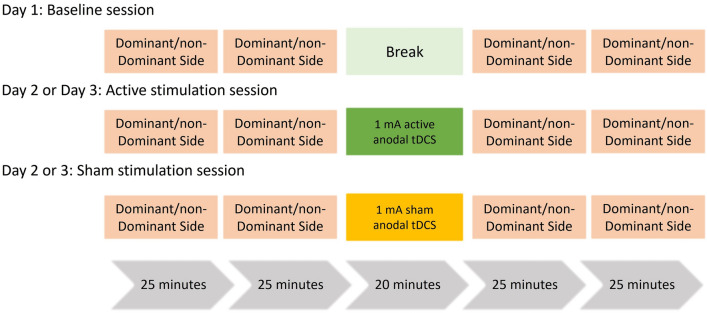
Presents an overview of the study design. Each session comprises two rounds of training involving both hands, with a break in between for session one. Sessions two and three receive either sham or active anodal tDCS between the two round of training.

The study consisted of three sessions, each conducted on separate days, with 48 h intervals in between sessions to wash out potential carryover effects of tDCS. Participant were randomized to either dominant or non-dominant hand, and for sham or active tDCS, leading to four different groups. The study design is illustrated in Fig. 4.

On the first day (baseline session), the participant was familiarized with the experimental setup and procedures. The first session consisted of two rounds of motor training with MPR on both sides, with a break of 20 min in between. On day two and three, participants were exposed to either sham or active tDCS in between the two rounds of motor training. All training sessions for a participant began with the same hand.

### Experimental setup

**Figure 5:**
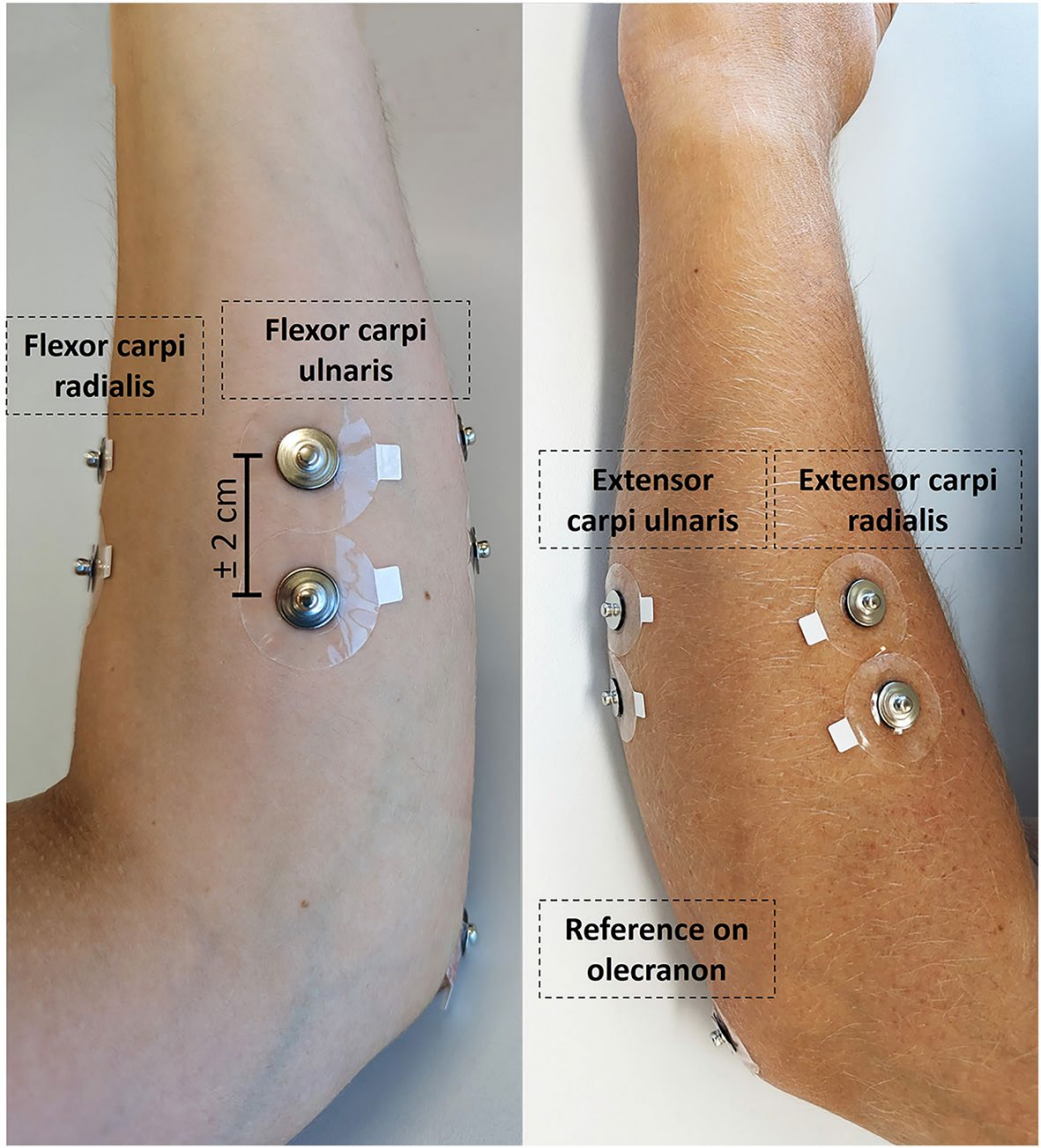
Surface electrode placement on one arm.

A total of eight bipolar self-adhesive electrodes, along with two reference Ag/AgCl electrodes, were positioned on both arms to record the EMG signals. The electrode diameter was 1 cm with an inter-electrode distance of approximately 2 cm. There were four bipolar electrodes per arm placed on the extensor carpi ulnaris, flexor carpi ulnaris, extensor carpi radialis, flexor carpi radialis muscles, and one reference electrode on the elbow. The positioning of the bipolar electrodes can be seen in Fig. 5 and was based on the orientation of the aforementioned muscles in wrist flexion/extension, elbow flexion/extension, wrist pro/supination and hand open/close movements, as described by previous work using BioPatRec, an open source platform for MPR^15^.

Working with one arm at a time, the electrodes were connected to an amplifier (ADS_BP4 ^43^) with embedded active filtering (high pass filter at 20 Hz and a low pass filter at 500 Hz) across four channels. The signals were amplified with a gain of 12 and sampled at 1000 Hz.

**Figure 6:**
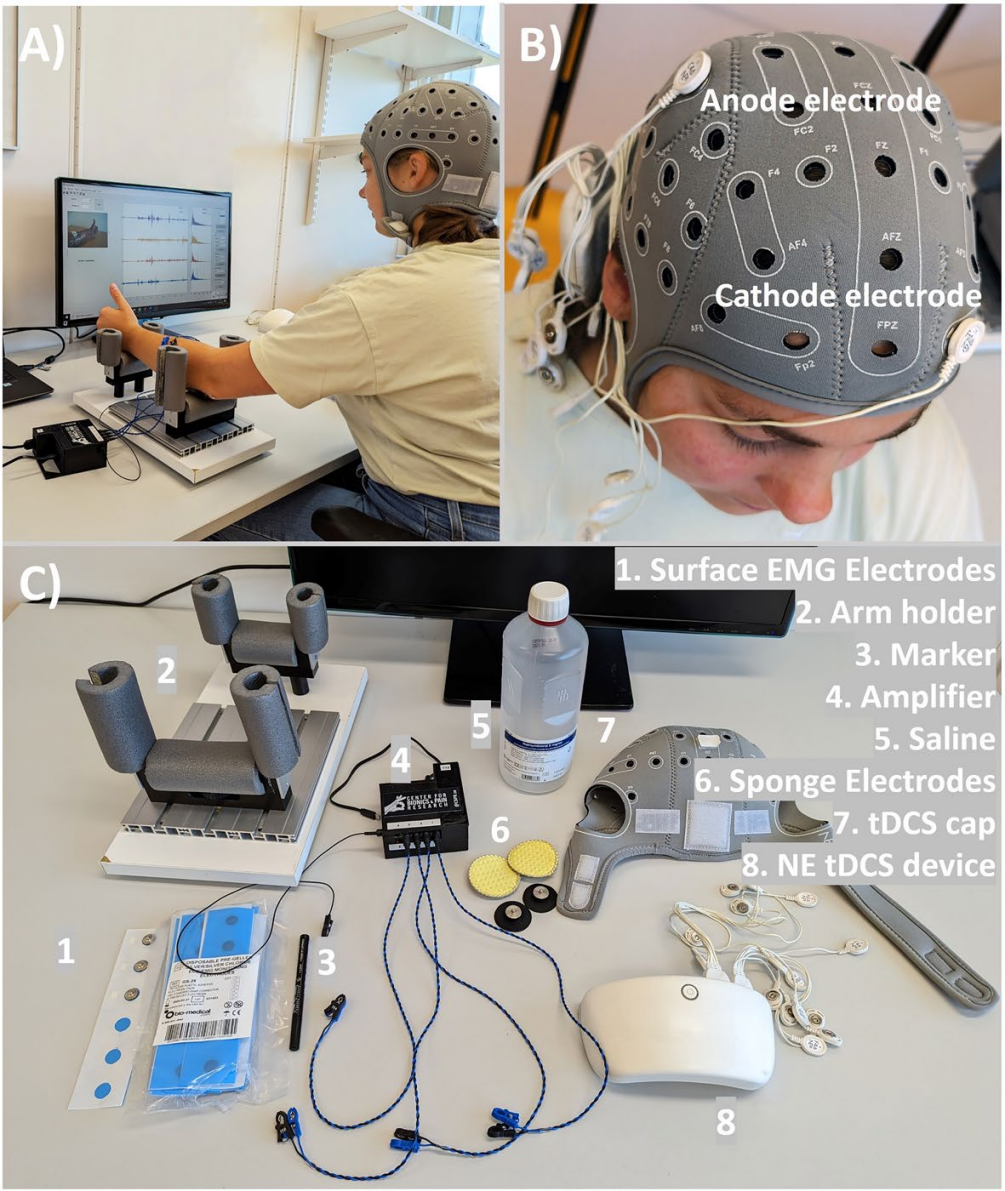
Setup of motor training (**A**), tDCS (**B**) and consumables and electrodes(**C**).

The setup of the motor training and the motion test can be seen in Fig. 6A. To stabilize the lower arm, it was positioned in a lower arm rest, supporting the elbow and wrist but enabling enough range to complete the movements. The quality of the EMG signals was assessed by conducting a short myoelectric recording of flexion, extension, open/close movements, and rest. All training was conducted within the BioPatRec environment^17^.

The motor training protocol involved movement recording and testing of 10 movements in the following order: hand open, hand close, hand flex, hand extend, supination, pronation, fine grip, side grip, thumb up, pointing with index finger, and state of no movement/relaxation. Each movement was recorded with one dummy repetition and three repetitions lasting for 3 s, with a three-second rest interval in between. After the 10 movements were recorded, four key signal features were extracted (mean absolute value, wavelength, zero crossings, and slope changes) to create the features vectors to train the classifier/decoder (linear discriminant analysis—LDA). LDA has been shown to be a successful decoder for this particular tasks, as outlined in the BioPatRec article^17^. The movement recording was immediately followed by a motion test, where participants were requested to perform all the trained movements in a randomized order: three trials of three repetitions per movement with 5 s time out. The motion test captured the executed movements to examine the performance of MPR in terms of completion rate, completion time, and real-time accuracy as described in the BioPatRec article^17^. After completing the movement recording and motion test on one hand, the process was replicated on the other hand, constituting a singular training round.

Following the completion of the first training round, a 20 min rest on day one was allocated before conducting the second round of the day (starting with the same hand). In days two and three, the break included 20 min of shame or active tDCS. The anodal tDCS (Fig. 6B) was applied on the non-dominant side, with an anode placed on the contralateral primary motor cortex (C4/C3) and the cathode over the ipsilateral prefrontal cortex (FP1/FP2), using a commercially available system (Starstim^®^ tES-EEG system). Through saline soaked 25 cm^2^ round sponge electrodes, a current of 1 mA was applied for a duration of 20 min, in between a 30 s ramp up- and down for the active tDCS. The ramp up and down happened both in the beginning and end in the sham tDCS, blinding the participant for the actual stimulation status. The baseline training session ended with marking the placement of the electrodes with a skin-friendly marker, to ensure that the electrodes were placed at exactly the same position on day two and three.

## Data Availability

The data that support the findings of this study are available upon reasonable request.
